# Indoxyl Sulfate-induced Vascular Calcification is mediated through Altered Notch Signaling Pathway in Vascular Smooth Muscle Cells

**DOI:** 10.7150/ijms.43184

**Published:** 2020-09-23

**Authors:** Kazutoshi Yamaguchi, Maimaiti Yisireyili, Sumie Goto, Katsuhiro Kato, Xian Wu Cheng, Takayuki Nakayama, Tadashi Matsushita, Toshimitsu Niwa, Toyoaki Murohara, Kyosuke Takeshita

**Affiliations:** 1 Department of Cardiology, Nagoya University Graduate School of Medicine, Nagoya, Japan.; 2 Biomedical Research Laboratories, Kureha Co., Tokyo, Japan.; 3 Department of Cardiology/Hypertension and Heart Center, Yanbian University Hospital, Yanji, Jilin, China.; 4 Department of Community Health and Geriatrics, Nagoya University Graduate School of Medicine, Nagoya, Japan.; 5 Department of Blood Transfusion, Aichi Medical University Hospital, Nagakute, Japan.; 6 Department of Clinical Laboratory, Nagoya University Hospital, Nagoya, Japan.; 7 Department of Blood Transfusion, Nagoya University Hospital, Nagoya, Japan.; 8 Shubun University, Ichinomiya, Aichi, Japan.; 9 Department of Clinical Laboratory, Saitama Medical Centre, Saitama Medical University, Kawagoe, Japan.

**Keywords:** keyword 1 indoxyl sulfate, keyword 2 vascular calcification, keyword 3 Notch signal.

## Abstract

**Introduction:** The aim of this study was to determine the role of Notch in indoxyl sulfate (IS)-induced vascular calcification (VC).

**Materials and methods:** VC and expression of Notch-related and osteogenic molecules were examined in Dahl salt-sensitive (DS), DS hypertensive (DH), and DH IS-treated rats (DH+IS). The effects of IS on expression of Notch receptors, apoptotic activity, and calcification were examined in cultured aortic smooth muscle cells (SMCs).

**Results:** Medial calcification was noted only in aortas and coronary arteries of DH+IS rats. Notch1, Notch3, and Hes-1 were expressed in aortic SMCs of all rats, but only weakly in the central areas of the media and around the calcified lesions in DH+IS rats. RT-PCR and western blotting of DH+IS rat aortas showed downregulation of Notch ligands, Notch1 and Notch3, downstream transcriptional factors, and SM22, and conversely, overexpression of osteogenic markers. Expression of Notch1 and Notch3 in aortic SMCs was highest in incubation under 500 μM IS for 24hrs, and then decreased time- and dose-dependently. Coupled with this decrease, IS increased caspase 3/7 activity and TUNEL-positive aortic SMCs. In addition, pharmacological Notch signal inhibition with DAPT induced apoptosis in aortic SMCs. ZVAD, a caspase inhibitor abrogated IS-induced and DAPT-induced *in vitro* vascular calcification. Knockdown of Notch1 and Notch3 cooperatively increased expression of osteogenic transcriptional factors and decreased expression of SM22.

**Conclusion:** Our results suggested that IS-induced VC is mediated through suppression of Notch activity in aortic SMCs, induction of osteogenic differentiation and apoptosis.

## Introduction

Indoxyl sulfate (IS) is a protein-bound uremic toxin, produced from metabolic conversion of dietary tryptophan, with vascular toxicity. Deterioration of renal dysfunction in chronic kidney disease (CKD) ultimately results in accumulation of IS. IS affects various signal pathways, such as those associated with oxidative stress, inflammation, cellular phenotype, and cell survival in vascular smooth muscle cells (VSMCs), and enhances vascular calcification (VC) 1. The cardiovascular mortality and morbidity rates are higher in patients with CKD compared with those of the general population 1. The severity of abdominal aortic calcification is associated with high risk of cardiovascular morbidity and mortality based on the high potential of cardiovascular events in patients with CKD 2. In patients with CKD, high IS plasma levels correlate with the progression of carotid artery atherosclerosis and cardiovascular events 3. Thus, it seems that IS is a risk factor for cardiovascular events in patients with CKD by inducing vascular injury and severe VC.

Notch signaling is a highly conserved signaling pathway associated with cellular activity, survival and differentiation 4. Notch receptors (Notch1-4) and their ligands (Jagged1-2 and Delta-like-1,3,4) are families of transmembrane proteins with large extracellular domains 5. Interaction of Notch receptors with membrane-bound ligands on the surface of neighboring cells leads to γ-secretase-dependent cleavage of the Notch intracellular domain (NICD) 5-7. These events result in the release of NICD into the nucleus, which interacts with the RBP-J protein, and the resultant complex functions as a transcription factor for various downstream target genes, such as hairy enhancer of split homolog-1 (Hes-1) and Hairy/enhancer-of-split related with YRPW motif protein 1 (Hey-1) 8 9. Notch1 and 3 receptors are highly expressed in VSMCs, and Notch1, rather than Notch3, mediates VSMC proliferation, migration and neointimal formation following vascular injury 8. In human atherosclerotic lesions, Notch1 is also highly expressed in the VSMCs in the media 10. Various inflammatory cytokines, such as interlukin-1β and tumor necrosis factors, increase the expression levels of Notch receptors, ligands, and downstream transcriptional factors to activate Notch signaling 11. On the other hand, IS-related cytotoxicity can alter Notch signal activity through various inflammatory mediators and reactive oxygen species 12.

VSMCs differentiate into osteoblast-like cells to mediate the deposition of bone matrix in the vascular wall 13. Bone-related transcriptional factors, such as Msx2, Sox9, and Runx2, which upregulate bone and chondrocyte proteins, have been detected in the calcified lesions on the vascular wall 13. These transcription factors regulate key processes important for osteoblast differentiation. Notch signaling is likely to be involved in this process based on its pleiotropic effects to determine cell fate. Notch target genes, Hes-1 and Hey-1, suppress Runx2 to inhibit osteoblastic differentiation 14. Conversely, pharmacological inhibition of Notch signaling or decrease in Notch signal activity induced by cell senescence, promotes osteogenic differentiation in cultured stromal cells 15. Apoptosis of VSMCs also plays a critical role in VC since apoptotic bodies released from VSMCs, as well as matrix vesicles, can concentrate and crystalize calcium and phosphate in the process of mineralization 13. As described above, Notch signaling also plays critical roles in cell survival. Haploinsufficiency of Notch1 promotes apoptosis of VSMCs in *ex vivo* culture conditions and murine models of vascular injury 8. Furthermore, loss-of-function mutation in Notch1 promotes VSMC apoptosis 16 and severe valve calcification with derepressed Runx2 transcriptional activity 17. Conversely, overexpression of Notch1 and Notch3 in rat VSMCs resulted in a significant decrease in cell apoptosis in association with a decrease in the BAX:BCL-xL mRNA expression ratio 16. Thus, IS-induced modulation of Notch signal activity is likely to be involved in VC.

The aim of the present study was to determine the role of Notch signaling in IS-induced VC. Specifically, we investigated the effects of IS accumulation on Notch1 signaling in VSMCs of Dahl salt-sensitive normotensive rats (DS), Dahl salt-sensitive hypertensive control rats (DH), and Dahl salt-sensitive hypertensive IS-administered rats (DH+IS) 18. We also examined the relationship between VC and expression of Notch-related molecules. Furthermore, we examined IS-induced decline in Notch signal activity and its effects on vascular calcification via apoptosis and differentiation in cultured VSMCs. The results showed that IS reduced Notch signal activity to induce calcification via apoptotic body formation and osteogenic differentiation.

## Materials and Methods

### Animal Studies

Experimental rats were prepared as reported previously 18. Briefly, five-week-old Dahl salt-sensitive rats (Dahl-Iwai S (DS), n = 24) were purchased from Japan SLC, Inc. (Hamamatsu, Shizuoka, Japan), and fed powder rat chow (CE-2, Clea, Tokyo, Japan) for 1 week. Then, six of DS rats were fed chow (CE-2) with low-salt (0.3% NaCl) intake in water, while another group of 24 were fed chow (CE-2) with high-salt (2.0% NaCl) intake in water. At 7 weeks of age, the latter group of DS rats developed spontaneous hypertension with systolic blood pressure (BP) of more than 140 mmHg. The spontaneously hypertensive rats were divided into two groups: control rats and IS-administered rats. Thus, the rat groups used in this study consisted of (i) Dahl salt-sensitive normotensive rats (DS, n = 6) with intake of 0.3% NaCl in water, (ii) Dahl salt-sensitive hypertensive control rats (DH, n = 12) with intake of 2.0% NaCl in water, (iii) Dahl salt-sensitive hypertensive IS-administered rats (DH+IS, n = 12) with intake of 2.0% NaCl and 200 mg/kg of IS (Alfa Aesar, Lancashire, UK) in water, for 30 weeks. Blood pressure was measured at the tail using a pneumatic cuff and a sphygmomanometer for small animals (UR-5000, Ueda Avancer Co., Tokyo, Japan) 18. Creatinine and BUN levels were measured using a Beckman Synchron CX3 auto-analyzer 18. Serum IS levels were measured by high-performance liquid chromatography, using the method reported in detail previously 19. Serum calcium and phosphate levels, and lipid profiles were measured by standard methods 7, 18, 20.

## Reagents

The following reagents and antibodies were used in the present study: anti-DLL4, anti-Hes-1, and anti-Hey-1 (Abcam, Cambridge, UK), anti-Jagged1, anti-Notch1, and anti-Notch3 antibodies (Santa Cruz Biotechnology, Santa Cruz, CA), anti-β actin (Calbiochem, La Jolla, CA), anti-rabbit IgG horseradish peroxidase (HRP)-linked antibody and anti-mouse IgG HRP-linked antibody (Cell Signalling Technology, Beverly, MA). H&E staining kit and von Kossa staining kit were purchased from Abcam (Cambridge, UK). Human aortic SMCs were purchased from Cascade Biologics (Portland, OR). Rat aortic SMCs were purchased from Cell Applications Inc. (San Diego, CA). Dulbecco's modified Eagle's medium (D-MEM), fetal bovine serum (FBS), penicillin-streptomycin, and trypsin-EDTA solutions were purchased from Gibco (Invitrogen, Grand Island, NY). IS was purchased from Sigma Chemical (St. Louis, MO). Calcium deposition detection kit was purchased from Wako (Osaka, Japan).

### Histological and immunohistochemical analyses

Aortic and cardiac sections were processed into 5-μm thick sections and stained with H&E and von-Kossa staining, using standard histological procedures 21. Immunohistochemistry was performed according to the streptavidin-biotinylated peroxidase complex method using standard protocols as described in detail previously 7, 20. Aortic sections were processed and stained using anti-Notch1 antibody (dilution, 1:100), anti-Notch3 antibody (dilution, 1:100), and anti-Hes-1 antibody (dilution, 1:100).

### Quantitative real-time polymerase chain reaction (RT-PCR)

Total RNA extraction, reverse-transcription, and quantitative PCR were performed as described in detail previously 22. The primer sequences used in this study are listed in Table 1.

### Western blot analysis

Equal amounts of rat aorta samples (30 mg) from the rats of each group were homogenized and total protein concentration was measured using the Pierce BCA Protein Assay Kit (Thermo Scientific Inc., Billerica, MA). VSMCs were lysed in lysis buffer (65 mmol/L Tris-HCl (pH 6.8), 3.3% sodium dodecyl sulfate (SDS), 10% glycerol, 2.2% bromophenol blue). 10μg of protein from the aorta homogenates and cultured cells were separated by SDS-polyacrylamide gel electrophoresis and transferred onto polyvinylidene difluoride (PVDF) membranes (Immobilon-P, Millipore Bedford, MA). The membranes were incubated with antibodies directed against rabbit polyclonal anti-Jagged1 antibody (dilution, 1:1000), rabbit polyclonal anti-DLL4 antibody (dilution, 1:1000), rabbit polyclonal anti-Notch1 antibody (dilution, 1:1000), rabbit polyclonal anti-Notch3 antibody (dilution, 1:1000), rabbit polyclonal anti-Hes-1 antibody (dilution, 1:2000), rabbit polyclonal anti-Hey-1 antibody (dilution, 1:1000), respectively. Then, the membranes were further incubated with HRP-linked secondary antibody (dilution, 1:10000) at room temperature for 1 hr. After washing with TBS-T three times, protein expression was visualized using the enhanced Chemi-Lumi one system (Nacalai Tesque, Kyoto, Japan). The intensity of protein bands was normalized to the amount of β-actin (an internal control, dilution, 1:10000) and expressed as ratio (fold increase) of the control value.

### Cell Cultures

Rat and human aortic SMCs were maintained in D-MEM containing 10% FBS supplemented with 100 U/mL penicillin and 100 μg/mL streptomycin at standard cell culture condition (37°C under 5% CO_2_ humidified atmosphere). The medium was replaced every three days until confluence. Only cells between passages 2 to 8 were used for experiments. Rat and human aortic SMCs were incubated with 0-1000 μmol/L of IS and 0-20 μmol/L of N-S-phenyl-glycine-t-butyl ester (DAPT), a Notch signal inhibitor, for the indicated time periods (IS: 0-96 hrs, DAPT: 0-72 hrs).

### TUNEL assay and caspase 3/7 activity assay

Rat and human aortic SMCs were incubated with 0-1000 μmol/L of IS and 0-20 μmol/L of DAPT for the indicated time periods (0-72 hrs). Apoptosis was examined by TUNEL assay, using the apoptosis detection TUNEL kit and Caspase 3/7 assay kit and the protocol supplied by the manufacturer (MK-500, Takara, Japan). In brief, cluttered samples were lysed with the assay buffer, and centrifuged at 15,000 x *g* for 15 min at 4°C. The supernatant was collected and used for the assay. TUNEL-positive cells were counted in 10 randomly representative fields under light microscopy.

### Measurement of calcium deposition in aortic SMCs

Calcium deposition in human aortic SMCs was induced by inorganic phosphate (3 mM) and examined as described in detail previously 23. The cells were seeded at a density of 2×10^5^ cells/well on 12-well culture plate in D-MEM containing 10% FBS for 48 hour. The cells were pre-incubated with DAPT (20 μM) and ZVAD (100 μM) for 1 hr, then stimulated with Pi (3 mM) or IS (1000 μM) for 72 hrs. Thereafter, the cultured cells were washed 3 times in ice cold PBS, then fixed with 70% ethanol at room temperature for 1 hr, then treated with 5% sliver nitrate under UV light for 45 min. The culture plates were photographed under light microscopy (×400) with digital camera (DN100, E-600, Nikon; Tokyo). Finally, the plates were washed with distilled water, and then decalcified with 0.6 N HCl for 12 hrs, and then standards and assay buffers were added to the plates. Absorbance was measured at 570-650 nm using a microplate reader (DS Pharmacy Biomedical Co., Osaka). The values were corrected by the amount of total protein. Total protein concentration was measured using the Pierce BCA Protein Assay Kit.

### SiRNA transfection of aortic SMCs

The siRNA oligo targeting Notch1 (catalog no. s129952), Notch3 (catalog no. s132728) and negative control were purchased from Thermo Fisher Scientific (Waltham, MA). We transfected siRNA to the rat aortic SMCs according to the instruction manual of Lipofectamine RNAiMAX (Thermo Fisher Scientific).

### Statistical analysis

Data are expressed as mean±SD. Differences between groups were assessed by the Student's t-test. Differences in the quantitative data among groups were analyzed by Fisher's protected least significant differences (PLSD) test of one-way analysis of variances (ANOVA). Results were considered significant when P<0.05.

## Results

### Laboratory parameters of DS, DH, and DH+IS rats

Table 2 summarizes the serial changes in several laboratory parameters in the DS, DH, and DH+IS rats throughout the 32-week study. Systolic blood pressure (BP) in the DH and DH+IS rats was significantly higher than DS rats, but similar between DH and DH+IS rats. Serum and urine levels of IS were significantly higher in DH+IS rats than in the other groups. However, there were no significant differences in blood urea nitrogen (BUN), serum creatinine, serum calcium (Ca), serum phosphate (P), calcium phosphorus product, and serum lipid profile among the groups.

### IS promotes aortic calcification in hypertensive rats

**Figure 1:**
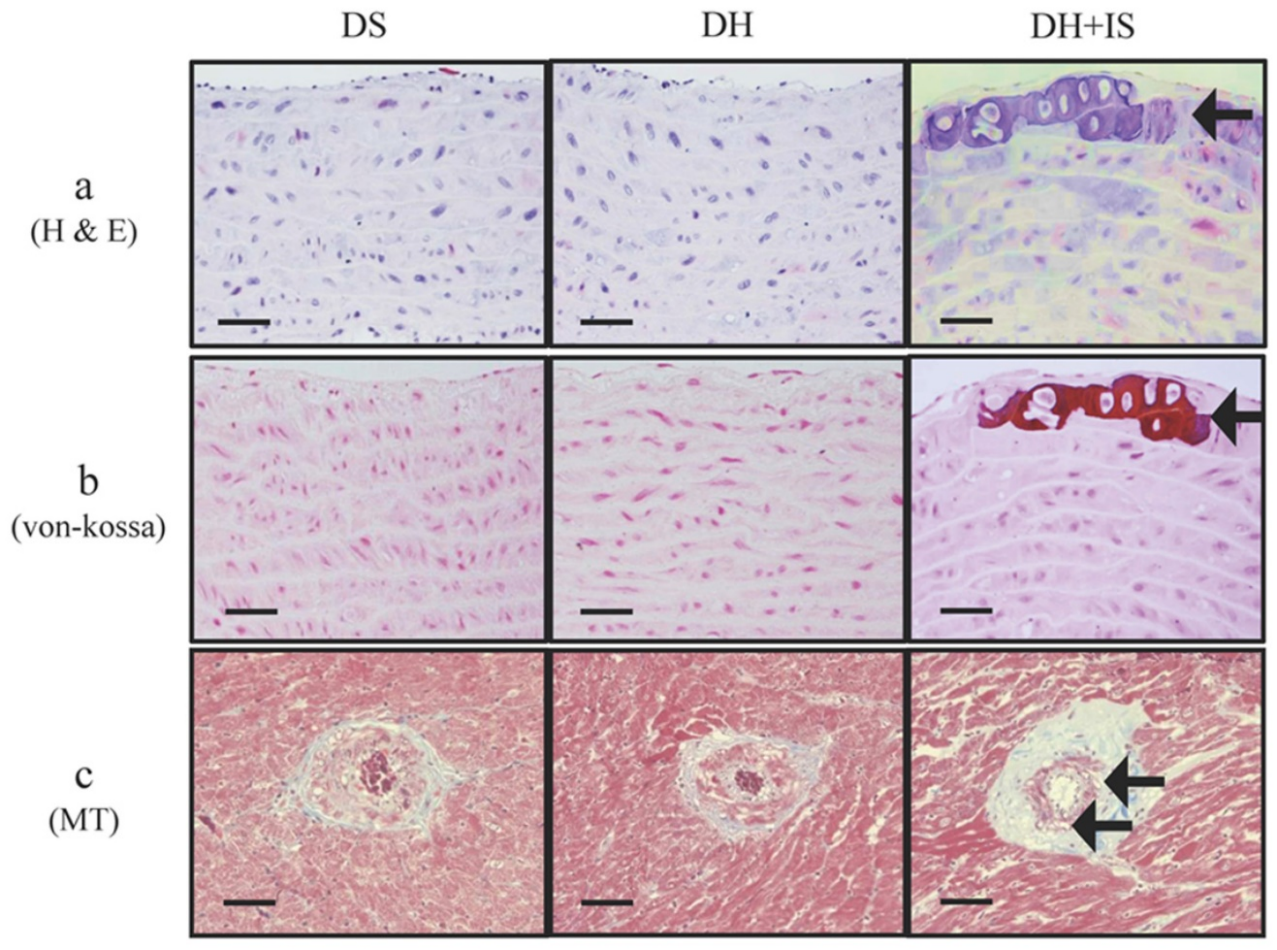
** IS promotes calcification in aortas and coronary arteries of Dahl-hypertensive rats.** H&E staining (a) and von-Kossa staining (b) of aortas from DS, DH and DH+IS rats. (c) Masson Trichrome staining (MT) of coronary arteries from DS, DH and DH+IS rat (c). (× 200 magnification, bar=50 µm). Arrows denote calcification.

Figure 1 shows medial calcific sclerosis in both the aortic arch and coronary arteries of DH+IS rats 18. These changes are similar to those observed in patients with Mönckeberg's arteriosclerosis 24. No calcified lesions were observed in the other groups.

### IS alters the expression of Notch receptors and Hes-1 in aortic SMCs

**Figure 2:**
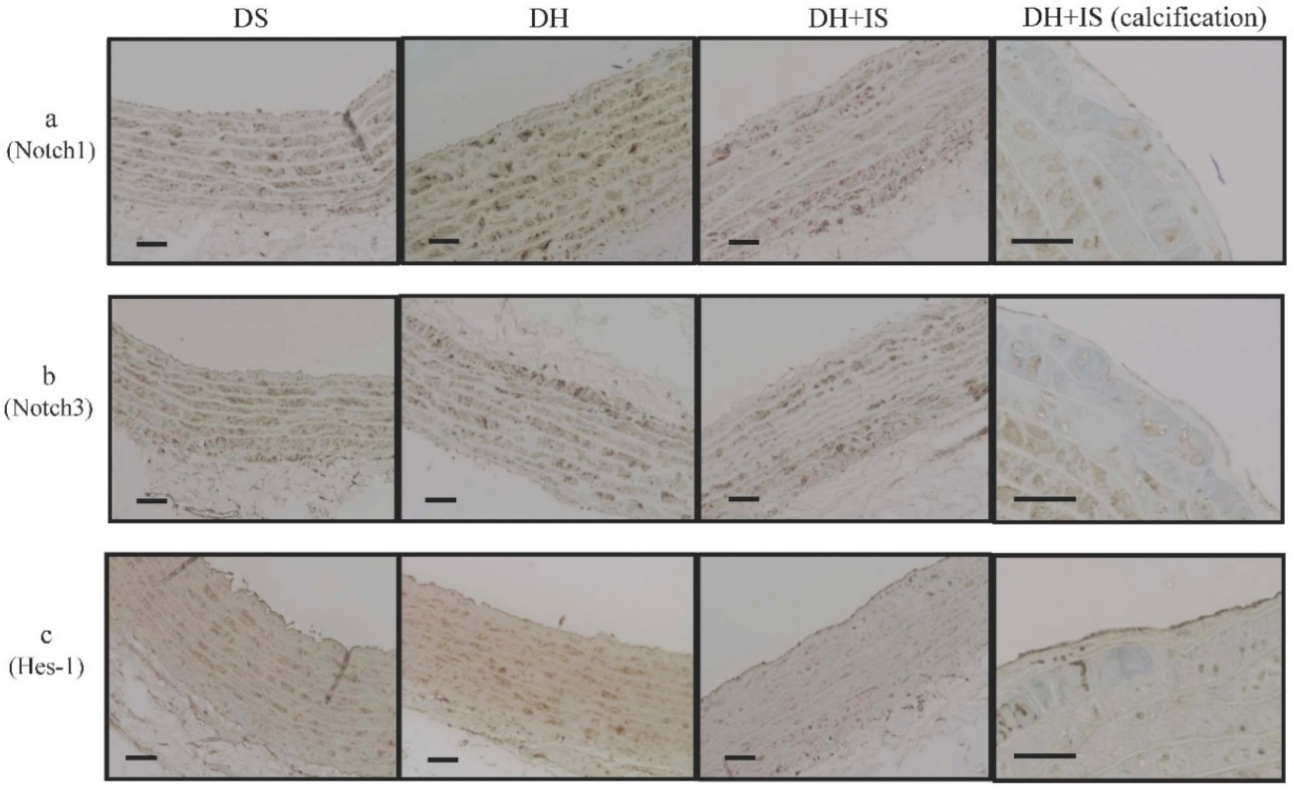
** IS reduces Notch Signal activity in Dahl-hypertensive rats.** Immunohistochemical analysis of Notch1 (a), Notch3 (b), and Hes-1 (c) in the aortas of DS, DH, and DH+IS rats (× 200 magnification, bar=50 µm), and calcification area of DH+IS rats (× 400 magnification, bar=50 µm).

Immunohistochemistry for Notch1, Notch3 and Hes-1 was applied to assess the expression of Notch receptors and Notch-related molecules, (Figure 2). Overexpression of Notch1 (Figure 2a), Notch3 (Figure 2b), and Hes-1 (Figure 2c) was noted in aortic SMCs from the DS and DH rats. However, the expression levels of these molecules were lower in aortic SMCs obtained from the central layer of the aortic media of DH+IS rats. Notably, signals for these molecules were hardly observed in the calcified lesions of DH+IS rats.

### IS reduces expression levels of Notch-related molecules in aorta

**Figure 3:**
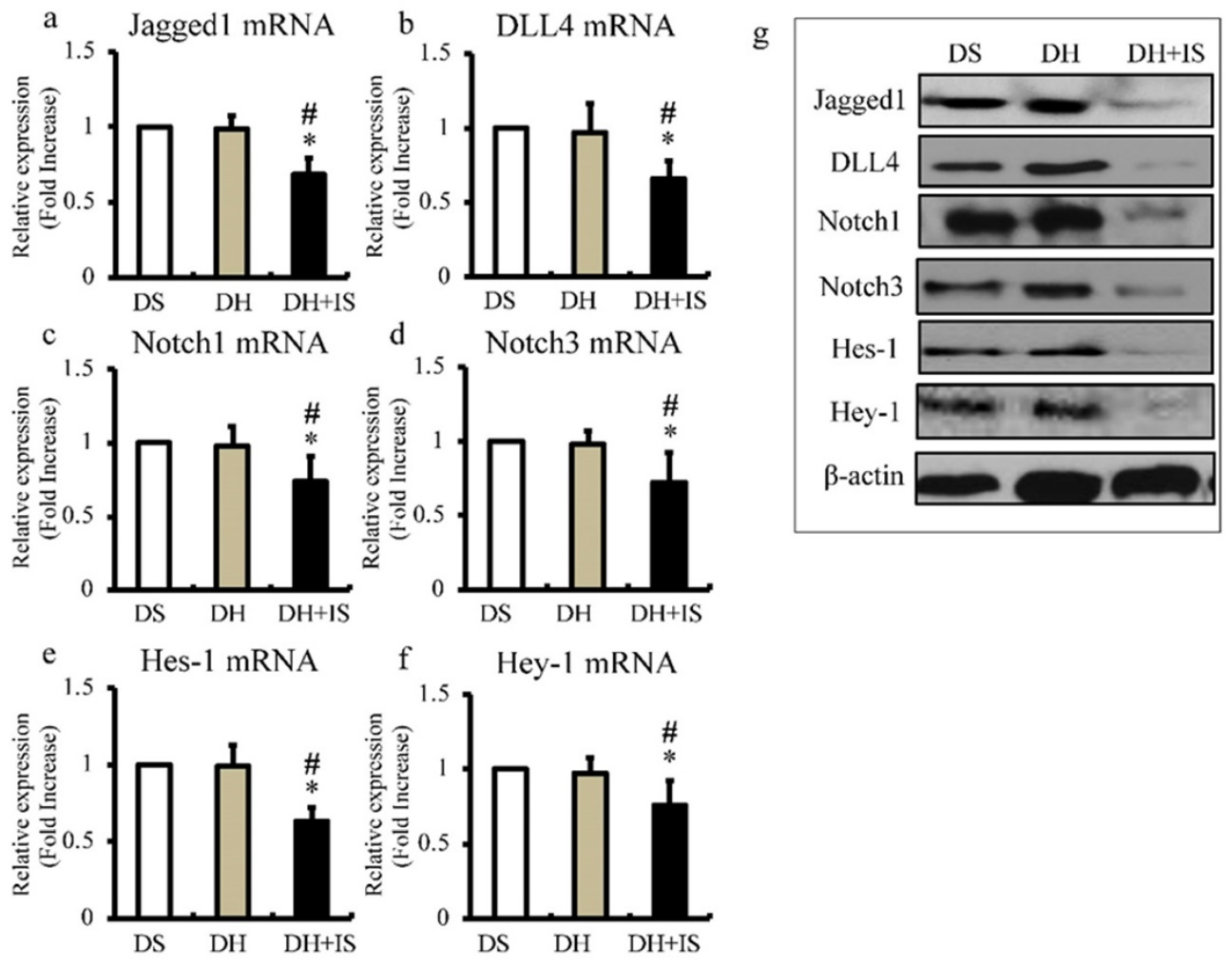
** IS reduces the expression of Notch-related molecules in Dahl-hypertensive rats.** Aortic mRNA and protein expression levels of Notch-related molecules in DS, DH, and DH+IS rats were analyzed by quantitative RT-PCR and western blotting; respectively. Values are mean ± SD (n=6-8). (a) Quantitative analysis of Jagged1 mRNA expression in aortic tissue. *p<0.0001 vs DS group, #p<0.0001 vs DH group. (b) Quantitative analysis of DLL4 mRNA expression in aortic tissue. *p<0.0007 vs DS group, #p<0.001 vs DH group. (c) Quantitative analysis of Notch1 mRNA expression in aortic tissue *p<0.005 vs DS group, #p<0.007 vs DH group. (d) Quantitative analysis of Notch3 mRNA expression in aortic tissue. *p<0.001 vs DS group, #p<0.003 vs DH group. (e) Quantitative analysis of Hes-1 mRNA expression in aortic tissue. *p<0.0001 vs DS group, #p<0.0001 vs DH group. (f) Quantitative analysis of Hey-1 mRNA expression in aortic tissue *p<0.004 vs DS group, #p<0.01 vs DH group. (g) Expression levels of representative proteins Jagged1, DLL4, Notch1, Notch3, Hes-1, and Hey-1 in the aorta of DS, DH, and DH+IS rats.

Next, we used quantitative RT-PCR to determine the expression levels of Notch ligands (Jagged1 and DLL4; Figure 3a and b), Notch receptors (Notch1 and Notch3; Figure 3c and d), and downstream transcriptional factors (Hes-1 and Hey-1; Figure 3e and f). In agreement with the results of immunohistochemistry (Figure 2), the mRNA expression levels of Notch-related molecules were lower in the aortas of the DH+IS rats, compared with the other groups. The results of western blot analysis shown in Figure 3g also demonstrated lower protein levels of Notch-related molecules in the aortas of the DS+IS rats.

### Involvement of IS in differentiation of aortic VSMCs into osteoblast-like phenotype

**Figure 4:**
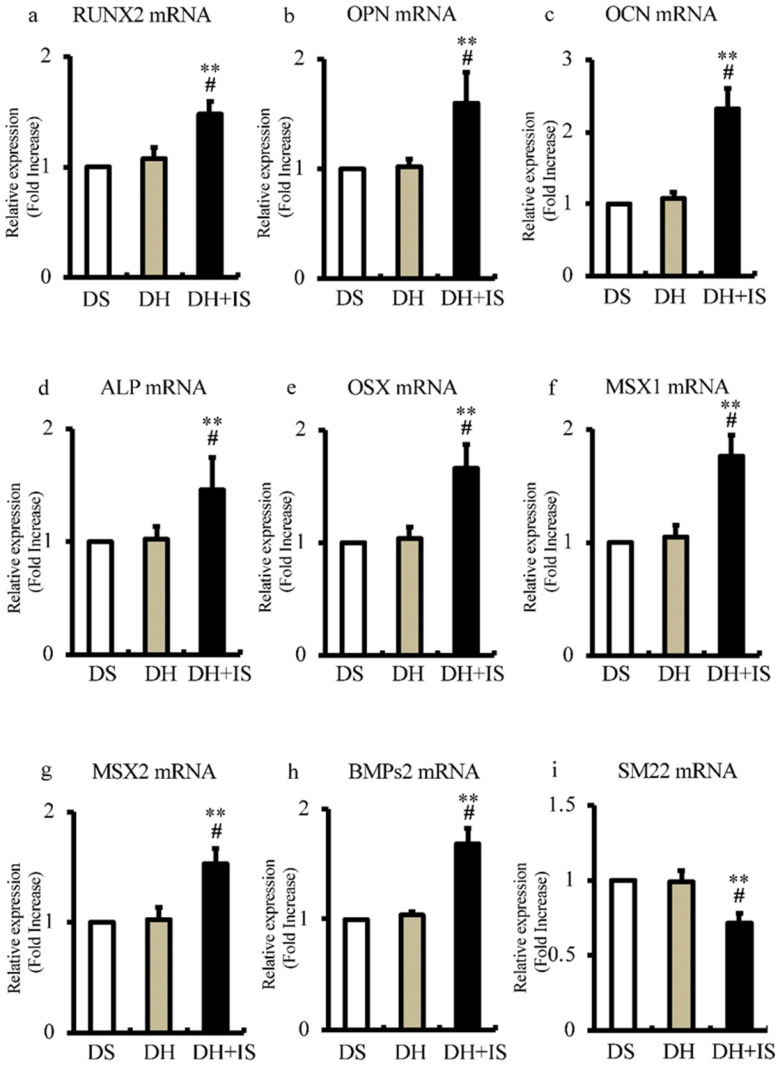
** IS increases the mRNA expression levels of osteoblast biomarkers and reduces that of SM22 in the aortas of Dahl-hypertensive rats.** Aortic mRNA expression levels of calcification biomarkers were analyzed in DS, DH, and DH+IS rats by quantitative RT-PCR. Data are mean ± SD (n=6-8 per group). (a) RUNX2 (**p<0.0001 vs DS group, #p<0.005 vs DH group), (b) osteopontin (OPN) (**p<0.0001 vs DS group, #p<0.0001 vs DH group), (c) osteocalcin (OCN) (**p<0.0001 vs DS group, #p<0.0002 vs DH group), (d) ALP (**p<0.0001 vs DS group, #p<0.0001 vs DH group), (e) osterix (OSX) (**p<0.0001 vs DS group, #p<0.0001 vs DH group), (f) MSX1 (**p<0.0001 vs DS group, #p<0.0001 vs DH group), (g) MSX2 (**p<0.0001 vs DS group, #p<0.0005 vs DH group), (h) BMPs2 (**p<0.0001 vs DS group, #p<0.0001 vs DH group), and (i) SM22 (**p<0.0001 vs DS group, #p<0.00015 vs DH group).

To determine the role of IS in the differentiation of aortic SMCs into an osteoblast-like phenotype, we analyzed the expression levels of bone-related transcriptional factors by RT-PCR. Figure 4 shows significantly higher mRNA expression levels of RUNX2, OPN, OCN, ALP, OSX, MSX1, MSX2, and BMPs2 in DH+IS rats, compared with the other groups. In contrast, the mRNA expression levels of SM22, a VSMC differentiation marker, was lower in DH+IS rats.

### IS alters expression levels of Notch1 and Notch3 in aortic SMCs

**Figure 5:**
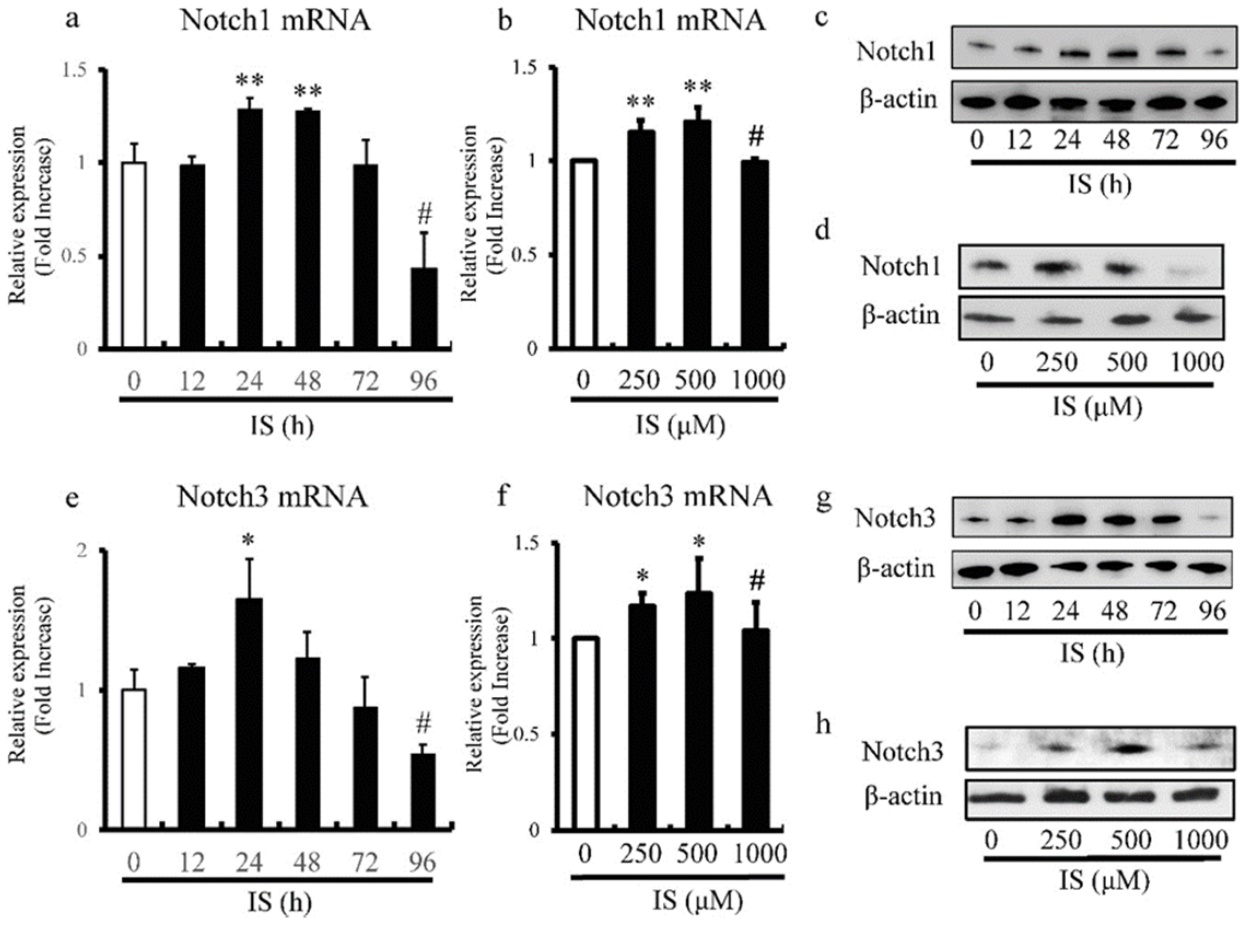
** IS reduces Notch1 and Notch3 expression in aortic smooth muscle cells (SMCs).** Rat and human aortic SMCs were treated with IS for the indicated time intervals (a and e) and at the indicated doses (b and f). (a) IS increased the expression level of Notch1 mRNA after 24- and 48-hr incubation but the level after incubation for 96 hrs was lower than non-treated control (**p<0.002 vs non-treated control, #p<0.0001 vs non-treated control). (b) IS increased Notch1 mRNA expression when used at 250 and 500 μmol/L, but the level in the presence of 1000 μmol/L was lower than at 250 and 500 μmol/L (**p<0.0002 vs non-treated control, #p<0.001 vs 500 μmol/L group). (e and f) A similar trend was noted for the effect of duration of incubation with IS (*p<0.01 vs non-treated control, #p<0.01 vs non-treated control) and dose of IS (*p<0.01 vs non-treated control, #p<0.05 vs 500 μmol/L group) on Notch3 mRNA. Data are mean ± SD (n=4-6 per group). (c, d, g, and h) Effects of duration of incubation and dose of IS on Notch1 and Notch3 protein levels in aortic SMCs cell lysates measured by western immunoblotting using anti-Notch1 and anti-Notch3 antibodies, respectively. IS decreased (c and d) Notch1 and (g and h) Notch3 protein levels after 96h and at the concentration of 1000 μmol/L. Representative data of 5 similar experiments.

We also examined the effects of different doses and duration of exposure to IS on the expression levels of Notch1 and Notch3 in cultured rat and human aortic SMCs by RT-PCR. The expression of Notch1 (Figure 5a) and Notch3 (Figure 5e) in rat aortic SMCs reached peak levels after 24h, and then decreased with time and exhibited significantly lower levels after 96h. Furthermore, the most significant increase and decrease in the expression levels of Notch1 (Figure 5b) and Notch3 (Figure 5f) in human aortic SMCs were noted at IS concentrations of 500 and 1000 μmol/L, respectively. Western blot analysis also showed decreased expression of Notch1 (Figure 5c) and Notch3 (Figure 5g) in rat aortic SMCs after 96h after exposure to IS. In addition, the most significant increase and decrease in the expression of Notch1 (Figure 5d) and Notch3 (Figure 5h) in human aortic SMCs were noted at IS concentration of 500 μmol/L and 1000 μmol/L.

### IS Induces apoptosis of aortic SMCs via Notch signal inhibition

**Figure 6:**
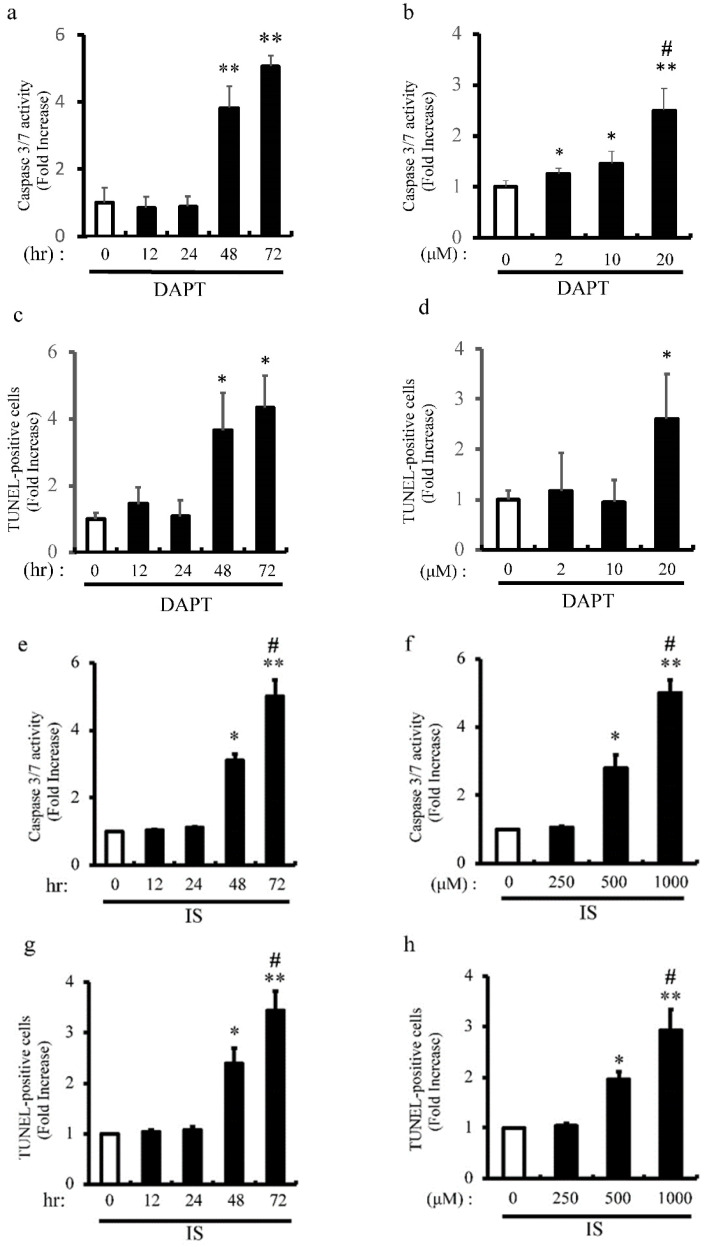
** IS and DAPT induce apoptosis of aortic SMCs.** Rat aortic SMCs were incubated with 0-20 μmol/L of N-S-phenyl-glycine-t-butyl ester (DAPT), a Notch signal inhibitor, for the indicated time periods (0-72 hour). Cellular apoptosis was detected by caspase 3/7 activity assay and TUNEL assay according to the protocol provided by the manufacturer. (a) Quantitative analysis of caspase 3/7 activity at different time points in rat aortic SMCs. **p<0.0001 vs non-treated control. Data are mean±SD (n=6 per group). (b) Quantitative analysis of caspase 3/7 activity at different dose in rat aortic SMCs. *p<0.01, **p<0.0001 vs non-treated control, #p<0.001 vs 10 μM group. Data are mean±SD (n=6 per group). (c) Quantitative analysis of TUNEL-positive cells at different time points in rat aortic SMCs. *p<0.001 vs non treated control. Data are mean±SD (n=6 per group). (d) Quantitative analysis of TUNEL-positive cells at different dose in rat aortic SMCs. *p<0.01 vs non-treated control. Data are mean±SD (n=6 per group). Human aortic SMCs were incubated with 0-1000 μmol/L of IS for the indicated time periods (0-72 hour). (e) Quantitative analysis of caspase 3/7 activity at different time points in human aortic SMCs. *p<0.001, **p<0.0001 vs non-treated control, #p<0.0001 vs 48 hr group. Data are mean±SD (n=6 per group). (f) Quantitative analysis of caspase 3/7 activity at different dose in human aortic SMCs. *p<0.003, **p<0.0001 vs non-treated control, #p<0.0001 vs 500 μM group. Data are mean±SD (n=6 per group). (g) Quantitative analysis of TUNEL-positive cells at different time points in human aortic SMCs. *p<0.001, **p<0.0001 vs non-treated control, #p<0.0003 vs 48 hr group. Data are mean±SD (n=6 per group). (h) Quantitative analysis of TUNEL-positive cells at different doses in human aortic SMCs. *p<0.0004, **p<0.0001 vs non treated control, #p<0.004 vs 500 μM group. Data are mean±SD (n=6 per group).

Previous our studies showed that pharmacological inhibition of Notch signaling and Notch1 haploinsufficiency promote apoptosis 6 8. We examined the proapoptotic effect of pharmacological inhibition of Notch signaling with DAPT on apoptosis of rat aortic SMCs (Figure 6a-d). Consistent with our previous studies, DAPT significantly activated caspase3/7 activity in dose- and time-dependent manners (Figure 6a and b). Consistently, apoptosis was time- and dose-dependently induced by DAPT in concordance with caspase3/7 activation as determined by TUNEL staining (Figure 6c and d). To examine the mechanism underlying IS-induced VSMCs apoptotic cell death, we examined the proapoptotic effect of IS on apoptosis in human aortic SMCs (Figure 6e-h). IS significantly activated caspase3/7 activity and increased TUNEL-positive cells dose- and time-dependently (Figure 6e-h). Considered together, these data indicate that IS induces apoptosis by decreasing Notch signaling.

### IS induces calcium deposition in aortic SMCs

**Figure 7:**
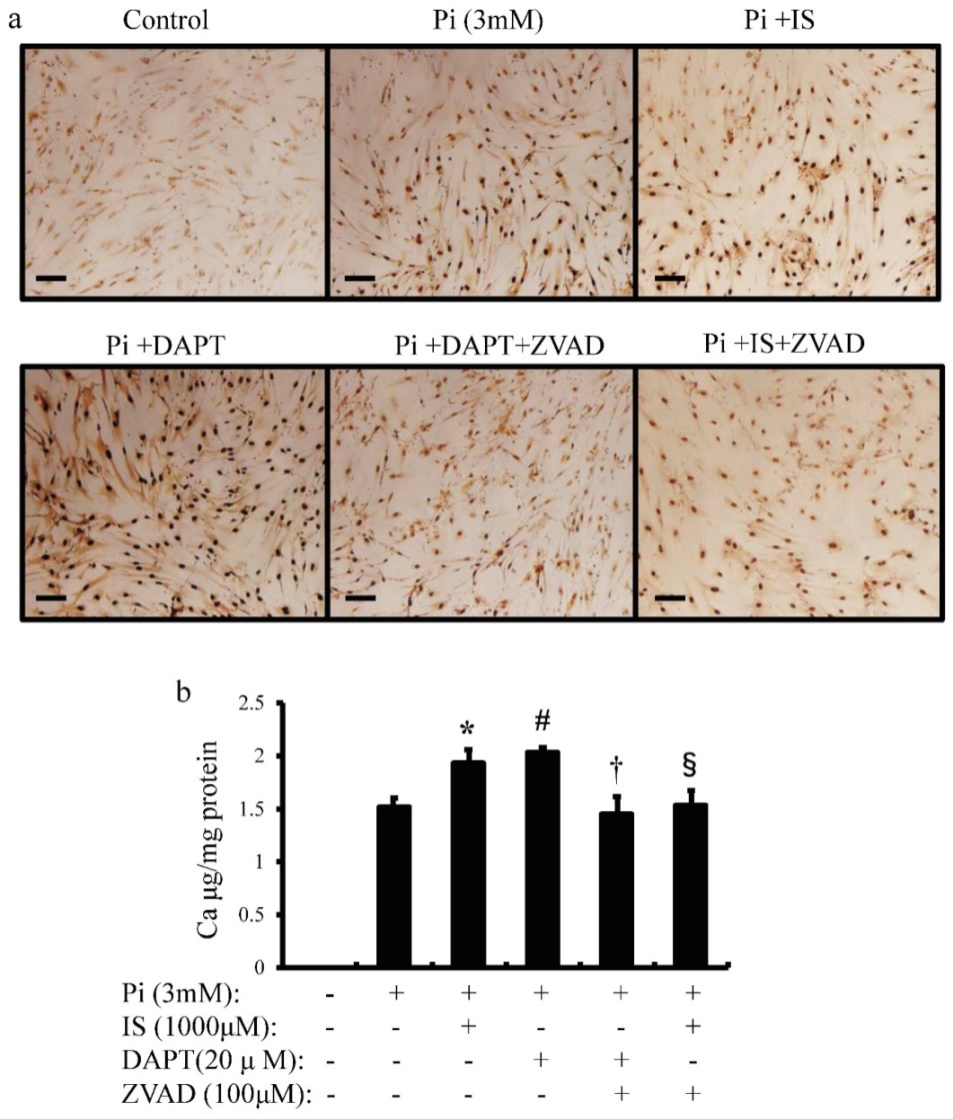

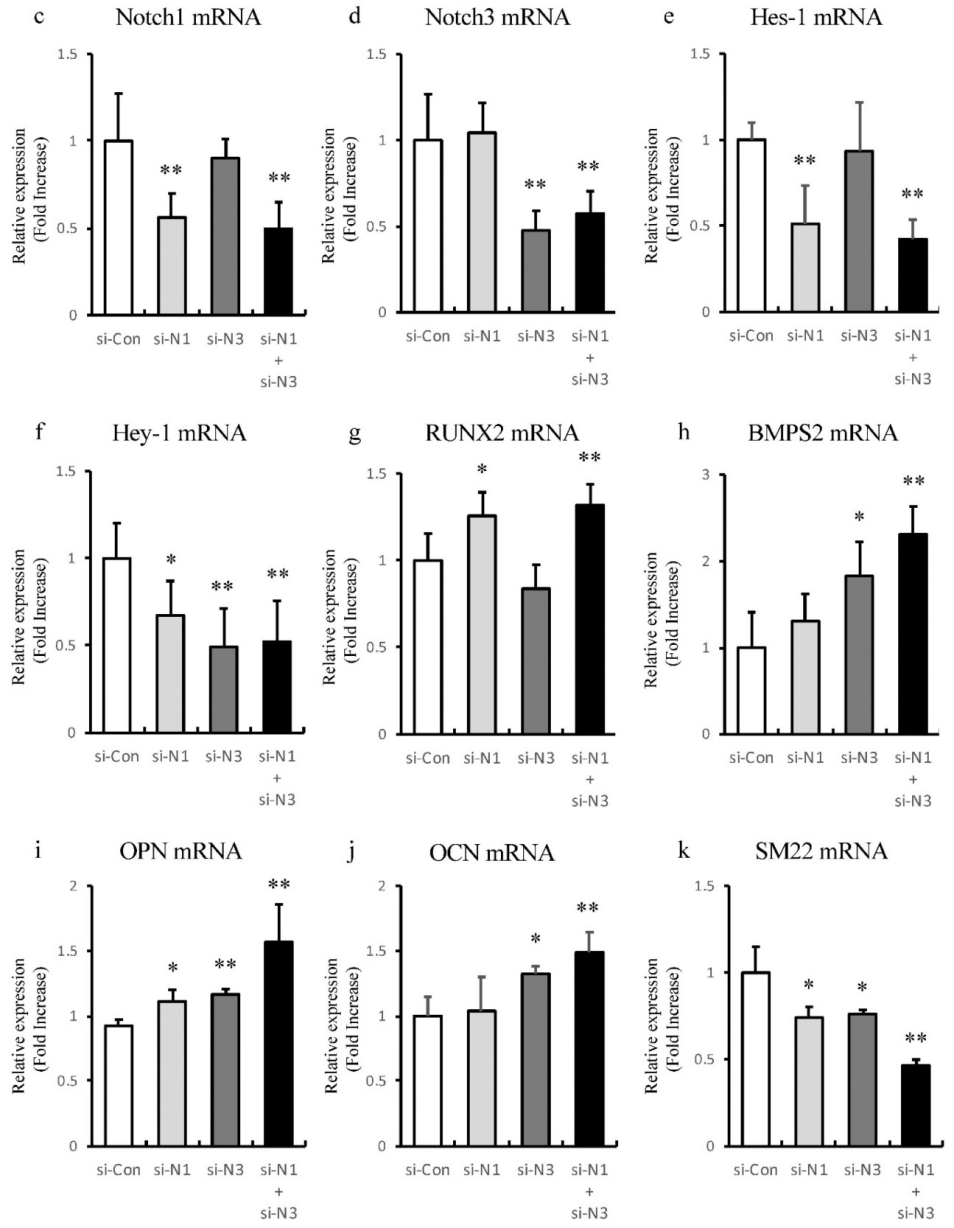
** IS induces cellular calcium deposition in aortic SMCs.** Calcium deposition in human aortic SMCs was induced by inorganic phosphate (3 mM). Cells were seeded at a density of 2×105 cells/well on 12-well culture plate in D-MEM containing 10% FBS for 48 hrs. Cells were pre-incubated with DAPT (20 μM) and ZVAD (100 μM) for 1 hr, then stimulated with Pi (3 mM) or IS (1000 μM) for 72 hrs. Absorbance was measured at 570~650 nm using a microplate reader. (a) Representative images showing calcium deposition in IS- or Pi-treated human aortic SMCs (×200 magnification, bar=50 µm). (b) Quantitative analysis of calcium deposition in IS- and Pi-treated human aortic SMCs. Data are mean±SD (n=6 per group). *p<0.009 vs Pi (3 mM) group, #p<0.0008 vs Pi (3 mM) group, †p<0.0059 vs DAPT-treated group, § p<0.009 vs IS-treated group. Expression levels of Notch-related and osteogenic-related mRNA with single knockdown of Notch1 and 3 or double knockdown of Notch1/Notch3 in rat aortic SMCs was analyzed by quantitative RT-PCR. Data are mean ± SD (n=6-8 per group). (c) Notch1 (**p<0.01 vs si-control group) (d) Notch3 (**p<0.01 vs si-control group) (e) Hes-1 (**p<0.01 vs si-control group) (f) Hey-1 (*p<0.05 vs si-control group, **p<0.01 vs si-control group) (g) Runx2 (*p<0.05 vs si-control group, **p<0.01 vs si-control group) (h) BMPS2 (*p<0.05 vs si-control group, **p<0.01 vs si-control group) (i) osteopontin (OPN) (*p<0.05 vs si-control group, **p<0.01 vs si-control group) (j) osteocalcin (OCN) (*p<0.05 vs si-control group, **p<0.01 vs si-control group), and (k) SM22 (*p<0.05 vs si-control group, **p<0.01 vs si-control group).

To examine whether IS-induced apoptosis in aortic SMCs promotes vascular calcification, we examined the effects of IS on inorganic phosphorus-induced calcium deposition (Figure 7a and b). Calcium deposition in cultured human aortic SMCs was examined microscopically (Figure 7a) and measured quantitatively with correction for protein concentrations (Figure 7b). Exposure to IS increased calcium deposition significantly. Pharmacological Notch signal inhibition with DAPT also increased calcium deposition. On the other hand, ZVAD, a caspase 9-specific inhibitor, significantly reduced calcium deposition in both IS-treated and DAPT-treated human aortic SMCs. These results suggest that IS induces calcification of the aorta through apoptosis of aortic SMCs.

### Knockdown of Notch receptors induces transdifferentiating of VSMCs into osteoblast-like phenotype

To find out whether Notch1 or Notch3 is involved in alteration of osteoblast-like and smooth muscle cell-like transcriptional factors, single knockdown of Notch1 and 3 or double knockdown of Notch1/Notch3 of rat aortic SMCs was performed to analyze the expression of Notch related genes and these transcriptional factors with RT-PCR (Figure 7c-k). Knockdown of Notch1 and Notch3 showed no significant redundant interaction between Notch1 and Notch3 (Figure 7c and d). Knockdown of Notch1 suppressed Hes-1 and Hey-1 to upregulate Runx2 and OPN (Figure 7e-g and i). Knockdown of Notch3 suppressed Hey-1, and increased BMPs2 and OCN (Figure 7f, h and j). On the other hand, single knockdown of both Notch1 and 3 suppressed the VSMC differentiation marker, SM22, respectively (Figure 7k). Synergic effects were observed in double knockdown of Notch1/Notch3 (Figure 7c-k). Thus, these dates indicate that inhibition of Notch1 and Notch3 cooperatively induces osteogenic transcriptional factors and suppresses smooth muscle cell-like transcriptional factor, SM22.

## Discussion

The novel findings of this study were that IS induced calcification of the aorta and reduced Notch signal activity in the central layer of the media layer as well as around the calcified lesions in hypertensive rats. The results also showed that the oral administration of IS for 30 weeks was followed by the appearance of calcified lesions in the media layer of both the aortic arch and coronary arteries (Figure 1) in the absence of any change in BP, renal function, and calcium-phosphorus product (Table 1). IS-induced decrease in Notch signal activity promoted osteogenic differentiation and apoptosis and ultimately the formation of calcified lesions. Immunohistochemical analysis demonstrated decreased expression of Notch1, Notch3, and Hes-1 in the central layer of the aortic media, which is a common site for calcification, and around calcified lesions (Figure 2).

Quantitative RT-PCR showed IS downregulated the expression of Notch ligands (Jagged1 and DLL4), Notch receptors (Notch1 and Notch3), and downstream transcriptional factors (Hes-1 and Hey-1) (Figure 3). Concordant with the decrease in Notch activity, IS also upregulated the expression of several osteogenic markers in the aorta and downregulated the VSMC differentiation marker SM22 in hypertensive rats (Figure 4). Notch signal inhibition promotes apoptosis 8 and apoptotic bodies from VSMCs promote crystallization of calcium and phosphate in preparation for mineralization 13. IS reduced Notch signaling in cultured aortic SMCs time- and dose-dependently (Figure 5). Coupled with the downregulation of Notch signaling, IS and pharmacological inhibition of Notch signaling similarly promoted apoptosis of aortic SMCs in the same manner (Figure 6). IS-induced calcification in cultured aortic SMCs was significantly suppressed by ZVAD, a caspase inhibitor (Figure 7). Finally, knockdown of Notch1 and Notch3 cooperatively increased the expression of osteoblast transcriptional markers and decreased VSMC differentiation marker, SM22.

Previous studies reported that administration of IS in hypertensive rats results in various pathological changes including medial calcification in both the aorta and coronary arteries, which is known in human as Mönckeberg's sclerosis 24. Mönckeberg medial sclerosis is specifically localized in both small transitional arteries, such as coronary arteries and large elastic-type arteries, such as the aorta 24 25, and it is frequently associated with accumulation of IS and severity of CKD 25. Animal models of Mönckeberg's sclerosis have been hardly discussed, but the IS-treated hypertensive rats is a suitable model to investigate the underlying pathophysiology. In patients with CKD, accumulation of uremic toxins (particularly inorganic phosphate, IS, advanced glycation end-products and inflammatory cytokines) is responsible for the high prevalence of vascular calcification 1. In this model, IS accumulation results in dysfunction of multiple cell signaling pathways 12. Notch signal is one of the most notable candidate signal affected by IS based on our finding of IS-induced downregulation of Notch-related molecules in the aorta, especially in the calcified lesions and the central layer of the aortic media, which is a common lesion of vascular calcification.

Little is known about the mechanisms of Notch downregulation. It is possible that IS-induced downregulation of Notch reflects abnormal response to tissue hypoxia since reduced Notch signaling was observed in the central layer of the aortic media, which is a common lesion in hypoxic damage 26. In this regard, IS is reported to activate aryl hydrocarbon receptor (AhR), which suppresses nuclear accumulation of the hypoxia-inducible factor (HIF)-α-AhR nuclear translocator (ARNT) complex, accompanied by an increase in the AhR-ARNT complex in the nucleus, resulting in failure of HIF-α-dependent response to hypoxia 27. Interestingly, Notch signal is ordinarily activated under hypoxic conditions 28. HIF-α interacts with the NICD and stabilizes this complex formation to upregulate the Notch signal pathway 28.

Since HIF-α is required for hypoxia-induced Notch signal activation, IS-induced disturbance in HIF-α pathway could suppress Notch signaling. Notch signal activation triggers a positive feed-back mechanism to produce Notch-related molecules 6. Meanwhile, pharmacological inhibition of enzymatic cleavage with DAPT was reported to downregulate Notch-related molecules 6. A decrease in Notch signal activity per se would reduce the expression of Notch-related molecules. Thus, IS-induced abnormal response to hypoxia would initiate decreased Notch signal activity itself to provide the negative feedback.

Osteogenic differentiation is an important process in the Mönckeberg medial calcific sclerosis 1, 25. Examination of calcified human valves has demonstrated increased expression of various osteogenic markers, such as Runx2 29. In the present study, we also showed overexpression of various osteogenic markers and underexpression of the VSMC differentiation marker SM22, with the observed downregulation of Notch activity in the aortas of IS-treated hypertensive rats (Figure 4). Notch signal plays critical roles in VSMC differentiation into mature and osteoblastic phenotypes 16. Interestingly, IS suppressed expression of Notch1 and Notch3, followed by a transient activation (Figure 5). To examine whether decline in Notch signal enhances osteogenic property and dedifferentiation of VSMCs, knockdown study of Notch1 and 3 was performed (Figure 7). No redundancy was observed between Notch1 and Notch3. In the downstream of Notch1 inhibition, increase in osteogenic transcription factors, Runx2 and OPN was observed 30 31. The mechanisms how Notch3 signal alters osteogenic transcription factors have been unclear. However, knockdown of Notch3 upregulated BMPs2 and OCN as previously shown 32. It has been reported that Notch pathway constitutes an instructive signal for SMC differentiation through an RBP-Jκ-dependent mechanism 33. Conversely, single knockdown of both Notch1 and 3 respectively suppressed the VSMC differentiation marker SM22. Synergically double knockdown of Notch1/3 strongly induced osteogenic transcription factors and VSMC dedifferentiation. Thus, Notch signal suppression with IS induces disarrangement of transcriptional factors to promote osteogenic property.

During development, Notch ligand, Jagged1, which is derived from endothelial cells, activates Notch signaling in VSMCs to promote VSMC maturation 34. In contrast, a decrease in Notch signal is reported to play a role in osteogenic differentiation 29. Garg et al. 17 showed that Notch1 signaling suppresses Runx2 signaling via interaction of Runx2 with Hrt family proteins, such as Hes-1 and Hey-1, and that human haploinsufficient Notch1 mutation promotes osteogenic differentiation and calcium deposition in aortic valve interstitial cells. As shown above, endothelial cells play critical roles in the regulation of vascular Notch signaling 34. IS promotes endothelial dysfunction through the induction of oxidative stress and progressive inflammatory process 12. Endothelial nitric oxide (NO)-knockout mice model showed that endothelial dysfunction was associated with reduced Notch signal activity in valve interstitial cells and activation of Runx2 signal 35. Thus IS-induced vascular injury seems to attenuate vascular Notch signal to promote the Mönckeberg medial calcific sclerosis.

The present study showed that IS-induced Notch downregulation was associated with enhanced apoptosis of human aortic SMCs, an important process in vascular calcification. Previous studies stressed the importance of Notch signal in cell survival under the conditions of cell stress and vascular injury 8, and demonstrated that pharmacological inhibition of Notch signaling and Notch1 haploinsufficiency increase apoptosis. Other studies showed that free radicals induced by IS in VSMCs activate nuclear factor kappa-B (NF-κB) and p53 pathways to promote apoptosis 36. Others reported that Notch1 signaling protects cells against NF-κB- and p53-induced apoptosis through the activation of Akt pathway 37. The NICD, which is cleaved out and released into cytoplasm, binds to the mammalian target of rapamycin complex (mTORC) to activate AKT signaling 37. Similarly, the Notch target gene, Hes-1, reduces the expression levels of phosphatase and tensin homolog (PTEN) to disinhibit the PI3K-AKT-mTORC pathway and promote cell survival. Thus, Notch signal is indispensable in protection against IS-induced proapoptotic stimuli.

In patients with CKD, high IS plasma levels are involved in the progression of atherosclerosis and aortic calcification 3, 38. Aortic calcification and Mönckeberg's arteriosclerosis are high risks for cardiovascular morbidity and mortality based on the high potential of cardiovascular events in patients with CKD 2, 39. Thus, IS-induced arterial calcification is a potentially suitable therapeutic target for the prevention of cardiovascular events. Furthermore, since the decrease in Notch signaling is involved in IS-induced aortic calcification, Notch signal activators could be potentially useful therapeutically. We showed previously that pitavastatin activates endothelial Notch1 through Akt-dependent stimulation of γ-secretase 7. Endothelial Notch activators, such as pitavastatin, might maintain VSMC Notch signaling against IS-induced disturbance of Notch signal pathway.

## Figures and Tables

**Table 1:** Sequences of primers used for RT-PCR.

	Forward (5'-3')	Reverse (5'-3')
R Jagged1	CATCGAGAAACACGGAGC	TATCCATATCATCCTCTTCCACTT
R Dll4	TGCGGATAACCAACGACG	CCCACAAAGCCATAAGGAC
R Notch1	GGTGCGAGCGCAGTGAAGGA	CCCGCTGCTGCCCTCTTTCC
R Notch3	AGCGAGCATCCTTATTTGAC	TTGCTGGACTAGGCGTT
R Hes-1	GCTGCTACCCCAGCCAGTG	GCCTCTTCTCCATGATAGGCTTTG
R Hey-1	AGTGAGCTGGACGAGACCAT	CTGGGTACCAGCCTTCTCAG
R RUNX2	TCATTCAGTGACACCACCAGG	TGTAGGGGCTAAAGGCAAAA
R OPN	CCCATCTCAGAAGCAGAATCTT	GTCATGGCTTTCATTGGAGTTG
R OCN	GACAAGTCCCACACAGCAAC	GGACATGAAGGCTTTGTCAGA
R ALP	GAGATGGTATGGGCGTCTC	GTTGGTGTTGTACGTCTTGGA
R OSX	AGAAGCCATACACTGACCTTTC	GGTGGGTAGTCATTGGCATAG
R MSX1	TGACTTCTTTGCCACTCGGTG	CTATGTCAGGTGGTACATGCTG
R MSX2	CCTCGGTCAAGTCGGAAAATTC	CGTATATGGATGCTGCTTGCAG
R BMPs2	TGAACACAGCTGGTCTCAGG	ACCCCACATCACTGAAGTCC
R SM22	CACTGGGCAAAGATGACT	CCACTTCTCCCTGCTTACTC
R β-actin	CTAAGGCCAACCGTGAAAAG	TACATGGCTGGGGTGTTGA
H Notch1	ATCCTGATCCGGAACCGAG	CGTCGTGCCATCATGCAT
H Notch3	TTTGAGGGTCAGAATTGTGAAGTG	TCGGTGTCCTGGACAGTCG
H β-actin	AGAAAATCTGGCACCACACC	GTCTCAAACATGATCTGGG

**Table 2:**
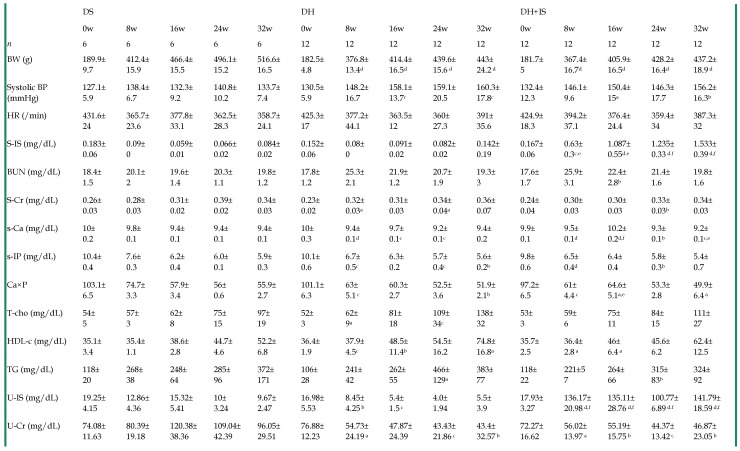
Time course of laboratory parameters in DS, DH and DH+IS rats.

Data are mean±SD. aP < 0.05; bP < 0.01; cP < 0.001; dP < 0.0001, compared with DS on the same week. eP < 0.01, fP <0.0001 compared with DH on the same week [by Fisher's LSD test (ANOVA)]. DS: Dahl salt-sensitive rats, DH: Dahl salt-sensitive hypertensive control rats with an intake of 2.0% NaCl in water, DH+IS: indoxyl sulfate-administered Dahl salt-sensitive hypertensive rats. BW: body weight, BP: blood pressure, S: serum, IS: indoxyl sulfate, BUN: blood urea nitrogen, Cr: creatinine, T-Cho: total cholesterol, HDL-C: high-density lipoprotein cholesterol, TG: triglyceride, U: urine.

## References

[1] Henaut L, Mary A, Chillon JM, Kamel S, Massy ZA (2018). The Impact of Uremic Toxins on Vascular Smooth Muscle Cell Function. Toxins.

[2] Furusawa K, Takeshita K, Suzuki S, Tatami Y, Morimoto R, Okumura T (2019). Assessment of abdominal aortic calcification by computed tomography for prediction of latent left ventricular stiffness and future cardiovascular risk in pre-dialysis patients with chronic kidney disease: A single center cross-sectional study. International journal of medical sciences.

[3] Sato B, Yoshikawa D, Ishii H, Kikuchi R, Arima T, Takeshita K (2013). Indoxyl sulfate, a uremic toxin, and carotid intima-media thickness in patients with coronary artery disease. International journal of cardiology.

[4] Baeten JT, Lilly B (2017). Notch Signaling in Vascular Smooth Muscle Cells. Advances in pharmacology.

[5] Radtke F, Wilson A, Mancini SJ, MacDonald HR (2004). Notch regulation of lymphocyte development and function. Nature immunology.

[6] Takeshita K, Satoh M, Ii M, Silver M, Limbourg FP, Mukai Y (2007). Critical role of endothelial Notch1 signaling in postnatal angiogenesis. Circulation research.

[7] Kikuchi R, Takeshita K, Uchida Y, Kondo M, Cheng XW, Nakayama T (2011). Pitavastatin-induced angiogenesis and arteriogenesis is mediated by Notch1 in a murine hindlimb ischemia model without induction of VEGF. Laboratory investigation; a journal of technical methods and pathology.

[8] Li Y, Takeshita K, Liu PY, Satoh M, Oyama N, Mukai Y (2009). Smooth muscle Notch1 mediates neointimal formation after vascular injury. Circulation.

[9] Fischer A, Schumacher N, Maier M, Sendtner M, Gessler M (2004). The Notch target genes Hey1 and Hey2 are required for embryonic vascular development. Genes & development.

[10] Tamai H, Kobayashi M, Takeshita K, Kodama A, Banno H, Narita H (2014). Possible involvement of Notch signaling in the pathogenesis of Buerger's disease. Surgery today.

[11] Shang Y, Smith S, Hu X (2016). Role of Notch signaling in regulating innate immunity and inflammation in health and disease. Protein & cell.

[12] Niwa T (2013). Targeting protein-bound uremic toxins in chronic kidney disease. Expert opinion on therapeutic targets.

[13] Leopold JA (2015). Vascular calcification: Mechanisms of vascular smooth muscle cell calcification. Trends in cardiovascular medicine.

[14] Zamurovic N, Cappellen D, Rohner D, Susa M (2004). Coordinated activation of notch, Wnt, and transforming growth factor-beta signaling pathways in bone morphogenic protein 2-induced osteogenesis. Notch target gene Hey1 inhibits mineralization and Runx2 transcriptional activity. The Journal of biological chemistry.

[15] Tang Z, Wei J, Yu Y, Zhang J, Liu L, Tang W (2016). gamma-Secretase inhibitor reverts the Notch signaling attenuation of osteogenic differentiation in aged bone marrow mesenchymal stem cells. Cell biology international.

[16] Harrison OJ, Visan AC, Moorjani N, Modi A, Salhiyyah K, Torrens C (2019). Defective NOTCH signaling drives increased vascular smooth muscle cell apoptosis and contractile differentiation in bicuspid aortic valve aortopathy: A review of the evidence and future directions. Trends in cardiovascular medicine.

[17] Garg V, Muth AN, Ransom JF, Schluterman MK, Barnes R, King IN (2005). Mutations in NOTCH1 cause aortic valve disease. Nature.

[18] Adijiang A, Goto S, Uramoto S, Nishijima F, Niwa T (2008). Indoxyl sulphate promotes aortic calcification with expression of osteoblast-specific proteins in hypertensive rats. Nephrology, dialysis, transplantation: official publication of the European Dialysis and Transplant Association - European Renal Association.

[19] Niwa T, Takeda N, Tatematsu A, Maeda K (1988). Accumulation of indoxyl sulfate, an inhibitor of drug-binding, in uremic serum as demonstrated by internal-surface reversed-phase liquid chromatography. Clinical chemistry.

[20] Yisireyili M, Takeshita K, Hayashi M, Wu H, Uchida Y, Yamamoto K (2016). Dipeptidyl peptidase- IV inhibitor alogliptin improves stress-induced insulin resistance and prothrombotic state in a murine model. Psychoneuroendocrinology.

[21] Aoyama T, Takeshita K, Kikuchi R, Yamamoto K, Cheng XW, Liao JK (2009). gamma-Secretase inhibitor reduces diet-induced atherosclerosis in apolipoprotein E-deficient mice. Biochemical and biophysical research communications.

[22] Uchida Y, Takeshita K, Yamamoto K, Kikuchi R, Nakayama T, Nomura M (2012). Stress augments insulin resistance and prothrombotic state: role of visceral adipose-derived monocyte chemoattractant protein-1. Diabetes.

[23] Chen NX, O'Neill KD, Duan D, Moe SM (2002). Phosphorus and uremic serum up-regulate osteopontin expression in vascular smooth muscle cells. Kidney international.

[24] McCullough PA, Agrawal V, Danielewicz E, Abela GS (2008). Accelerated atherosclerotic calcification and Monckeberg's sclerosis: a continuum of advanced vascular pathology in chronic kidney disease. Clinical journal of the American Society of Nephrology: CJASN.

[25] Lanzer P, Boehm M, Sorribas V, Thiriet M, Janzen J, Zeller T (2014). Medial vascular calcification revisited: review and perspectives. European heart journal.

[26] Billaud M, Hill JC, Richards TD, Gleason TG, Phillippi JA (2018). Medial Hypoxia and Adventitial Vasa Vasorum Remodeling in Human Ascending Aortic Aneurysm. Frontiers in cardiovascular medicine.

[27] Asai H, Hirata J, Hirano A, Hirai K, Seki S, Watanabe-Akanuma M (2016). Activation of aryl hydrocarbon receptor mediates suppression of hypoxia-inducible factor-dependent erythropoietin expression by indoxyl sulfate. American journal of physiology Cell physiology.

[28] Landor SK, Lendahl U (2017). The interplay between the cellular hypoxic response and Notch signaling. Experimental cell research.

[29] Garg V (2016). Notch Signaling in Aortic Valve Development and Disease. In: Nakanishi T, Markwald RR, Baldwin HS, Keller BB, Srivastava D, Yamagishi H, editors. Etiology and Morphogenesis of Congenital Heart Disease: From Gene Function and Cellular Interaction to Morphology. Tokyo.

[30] Hilton MJ, Tu X, Wu X, Bai S, Zhao H, Kobayashi T (2008). Notch signaling maintains bone marrow mesenchymal progenitors by suppressing osteoblast differentiation. Nature medicine.

[31] Yao XX, Lu JB, Ye ZD, Zheng L, Wang Q, Lin ZQ Hairy/enhancer of Split Homologue-1 Suppresses Vascular Endothelial Growth Factor-induced Angiogenesis via Downregulation of Osteopontin Expression. 2017; 7: 898.

[32] Wang H, Jiang Z, Zhang J, Xie Z, Wang Y, Yang G (2017). Enhanced osteogenic differentiation of rat bone marrow mesenchymal stem cells on titanium substrates by inhibiting Notch3. Archives of oral biology.

[33] Doi H, Iso T, Sato H, Yamazaki M, Matsui H, Tanaka T (2006). Jagged1-selective notch signaling induces smooth muscle differentiation via a RBP-Jkappa-dependent pathway. The journal of biological chemistry.

[34] High FA, Lu MM, Pear WS, Loomes KM, Kaestner KH, Epstein JA (2008). Endothelial expression of the Notch ligand Jagged1 is required for vascular smooth muscle development. Proceedings of the National Academy of Sciences of the United States of America.

[35] Bosse K, Hans CP, Zhao N, Koenig SN, Huang N, Guggilam A (2013). Endothelial nitric oxide signaling regulates Notch1 in aortic valve disease. Journal of molecular and cellular cardiology.

[36] Niwa T, Shimizu H (2012). Indoxyl sulfate induces nephrovascular senescence. Journal of renal nutrition: the official journal of the Council on Renal Nutrition of the National Kidney Foundation.

[37] Zeng C, Xing R, Liu J, Xing F (2016). Role of CSL-dependent and independent Notch signaling pathways in cell apoptosis. Apoptosis: an international journal on programmed cell death.

[38] Gao H, Liu S (2017). Role of uremic toxin indoxyl sulfate in the progression of cardiovascular disease. Life sciences.

[39] Lehto S, Niskanen L, Suhonen M, Ronnemaa T, Laakso M (1996). Medial artery calcification. A neglected harbinger of cardiovascular complications in non-insulin-dependent diabetes mellitus. Arteriosclerosis, thrombosis, and vascular biology.

